# The expanded interactional model of exercise addiction

**DOI:** 10.1556/2006.2021.00061

**Published:** 2021-09-14

**Authors:** Jacob S. Dinardi, Alexei Y. Egorov, Attila Szabo

**Affiliations:** 1 Department of Kinesiology, San Francisco State University, San Francisco, CA, USA; 2 Department of Psychiatry and Addictions, Faculty of Medicine, St. Petersburg State University, St. Petersburg, Russia; 3 Laboratory of Behaviour Neurophysiology and Pathology I.M. Sechenov Institute of Evolutionary Physiology and Biochemistry of Russian Academy of Sciences, St. Petersburg, Russia; 4 Institute of Health Promotion and Sport Sciences, ELTE Eötvös Loránd University, Budapest, Hungary; 5 Institute of Psychology, ELTE Eötvös Loránd University, Budapest, Hungary

**Keywords:** addiction, compulsion, dependence, physical activity, training

## Abstract

**Background and aims:**

Cited in over 100 articles, the *interactional model of exercise addiction* (Egorov & Szabo, 2013) forms the theoretical foundation of many studies on the risk of exercise addiction. Still, the inclusion of previously omitted determinants could make it more useful. Therefore, this review presents the expanded version of the original model.

**Method:**

We added ‘*self-concept’* as another determinant in the ‘personal factors’ domain and ‘*attractive alternatives’* to the ‘situational factors’ domain. Further, we doubled the reasons for exercise in the ‘incentives for exercise domain.’ Last, we added a new domain, the ‘*exercise-related stressors*,’ to illustrate that exercise itself might be a source of stress.

**Results:**

The expanded model is more inclusive and accounts for a greater combination of interactions playing roles in exercise addiction. Overlooking the eventuality that stress resulting from exercise might also fuel the dysfunction was a significant omission from the original model, rectified in the current update. Finally, the new expansions make the model more applicable to competitive situations too

**Conclusion:**

The expanded interactional model of exercise addiction is more comprehensive than its original version. It also accounts for the exercise or sport-related stress as possible fuel in addictive exercise behavior.

Exercise and sports are beneficial aspects of people's lives (Bushman, 2019). The World Health Organization (WHO; Bull et al., 2020) and other institutions concerned with health and human services offer guidelines on the minimum levels of physical activity individuals should engage in to receive essential health benefits. The upper threshold at which physical activity remains beneficial and is safe has not been widely reported outside of examining certain long-term effects of athletic training, for example, O'Keefe et al. (2012), nor do general guidelines presently exist for maximum levels of physical activity participation. However, an individual's exercise behavior can become maladaptive, manifesting in compulsive exercise, exercise dependence, and exercise addiction. The latter term involves both compulsion and dependence (Goodman, 1990), but they are used interchangeably in the literature to denote the same problem.

We talk about exercise addiction when significant involvement in exercise behavior adversely affects a person's health, relationships, and productivity (de la Vega, Parastatidou, Ruíz-Barquín, & Szabo, 2016; Nogueira, Molinero, Salguero, & Márquez, 2018). Moreover, exercise addiction is marked by an unhealthy obsession with physical activity leading to compulsive behavior and the inability to regulate one's involvement with exercise or sports. While cases in scholastic writings are few, Juwono and Szabo (2020) have identified 100 cases on the internet. Currently, there are no diagnostic criteria for exercise addiction. Indeed, the dysfunction is unlisted in the latest edition of the Diagnostic and Statistical Manual of Mental Disorders (DSM-5; American Psychiatric Association, 2013) due to insufficient clinical evidence.

Although this maladaptive exercise behavior has been the subject of scholarly research for over 50 years, the disorder is challenging to characterize and, therefore, it remains unclear how many individuals are affected by exercise addiction as demonstrated by widely variable rates of prevalence reported in the extant literature. Cook, Hausenblas, and Freimuth (2014) summarizes a number of studies that reported prevalence of exercise compulsion, dependence, and addiction. Furthermore, Cook et al. (2014) identifies several of the challenges that exist in measuring the prevalence of exercise addiction which include the lack of a unified theoretical approach, a focus on symptom severity over cases of fully developed exercise addiction, and differences in sample group characteristics (i.e. competitive athletes, recreational athletes, exercisers) which lead to bias in prevalence estimates.

Several proposed theoretical approaches define and assess exercise addiction, most resembling those applied in assessing substance use disorders (i.e., Hausenblas & Downs, 2002). While such approaches are valid, the underlying assumption that exercise, as an object of addiction, is analogous to drugs or alcohol in substance use disorders (SUDs) raises some questions. For instance, are mood-modification, tolerance, or withdrawal symptoms useful indices in identifying exercise addiction? Alternately, are the tools used in studying SUDs also appropriate for studying exercise addiction? Importantly, do these approaches give proper consideration to the mindsets and goals of individuals who are training with sports-related performance in mind, in addition to individuals who are exercising to improve their health or appearance? Models of substance use disorders address fundamental issues of addiction; however, they have limited potential to account for more specific exercise-related behavior such as chasing goals to achieve an idealistic physique or ideal performance. Instead of relying on SUD models in the definition of exercise addiction, it may be more beneficial to focus on factors related to sports and exercise and the people who participate in them. Conceptualizing exercise addiction as a disorder with unique antecedents, contributing factors, and consequences that set it apart from other dysfunctions, much like gambling disorder has been conceptualized in the Diagnostic and Statistical Manual of Mental Disorders (DSM-5; American Psychiatric Association, 2013), could generate more precise ideas about what exercise addiction is and how to assess it.

Therefore, Egorov and Szabo (2013) have proposed the *interactional model* of exercise addiction to describe its etiology. This model describes only primary exercise addiction, which Veale (1995) describes as developing in the absence of eating disorders in contrast to secondary exercise addiction, in which certain eating disorders (i.e., anorexia, bulimia) are always present. Freimuth, Moniz, and Kim (2011) extend the concept of secondary exercise addiction by applying it to exercise behavior that is foremost motivated by the desire to relieve anxiety people experienced specifically as a result of not exercising (i.e. obsessive compulsive disorder). Whether body dysmorphic disorder should also be considered a primary disorder in the context of exercise addiction remains an open question. In brief, when a person's maladaptive exercise behavior is driven by motivation typically associated with dedicated exercise, it is considered primary exercise addiction; when the behavior is foremost driven by motivation to soothe another disorder, it is secondary exercise addiction. Thus, the model is concerned only with factors that lead an individual to be interested in exercise as an outlet for physical activity, their specific motivations, and orientations that describe their approaches in using exercise and sports to experience mastery, enhance specific elements of their life, and as means of stress-coping.

The model as originally conceptualized (Fig. 1) illustrates the pathways that individuals may come to experience exercise addiction through any of several courses. The first of these pathways results from personal and situational factors. The second results from negative stress-coping (escape), while the third recognizes that individuals' mastery orientation may drive them toward exercise addiction. The model also leaves room for the possibility that an individual can shift from exercise addiction back to healthy exercise. With this model, Egorov and Szabo have provided a comprehensive overview of antecedents of exercise and most related factors associated with the onset of exercise addiction. Still, over time, new determinants were identified, leaving room for the expansion of the model.

**Fig. 1.**
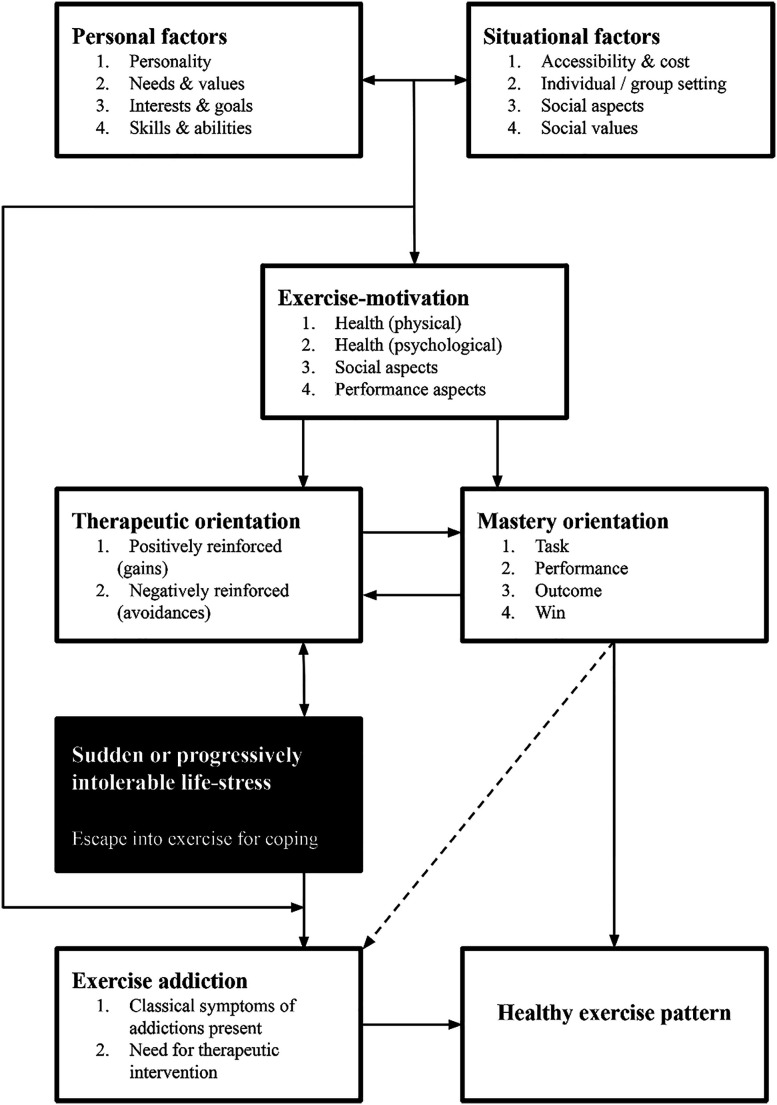
The interactional model of exercise addiction (Egorov & Szabo, 2013)

In pursuit of a better understanding of exercise addiction, we considered how the interactional model of exercise addiction may be expanded to be encompass additional factors that contribute to a person's exercise interests as well as factors inclusive of sports and training, which are often different than those strictly related to exercise and fitness. For example, the desire to enhance one's physical appearance or health are common motivations in exercise, while seeking improved sports performance, either for competitive reasons or to achieve personal goals, is associated with training (Frederick & Ryan, 1993; Reifsteck, Gill, & Labban, 2016). We posit that, among other reasons, exercise addiction has been challenging to assess because, as it is currently conceptualized, it must capture maladaptive behavior within the domain of exercise as well as the related domains of sports and training. In each of these domains, individual motivation can range widely and is constituted of different incentives as well as avoidances. While a parsimonious model is preferable in the examination of any behavior, understanding the unique courses that lead individuals into exercise addiction requires considering the broad contexts in which individuals are physically active and the distinct motivations underlying them that can become maladaptive during the exercise addiction process.

The original interactional model of exercise addiction, while comprehensive, did not consider certain factors that may play a significant role in exercising or sports becoming an unhealthy focus in a person's life. For example, among the personal factors, how people see and think about themselves could be a mediator of maladaptive exercise (Corazza et al., 2019). Choice alternatives can also influence one's gravitation toward and relationship with an activity (Lichtenstein, Christiansen, Elklit, Bilenberg, & Støving, 2014). Exercise motives also include enjoyment (Rodrigues, Teixeira, Neiva, Cid, & Monteiro, 2020), sensation seeking (Kotbagi, Morvan, Romo, & Kern, 2017), challenge (Youngman & Simpson, 2014), and less common reasons, such as strengthening family bonds (Wagoner, 2018) or exercising as an outlet to support one's sobriety and recovery from other addictions (Corbett & England, 2018). Apart from these exclusions, the original interactional model does not consider how exercise, sports, and training can become sources of stress that further fuel the addiction.

This review expands the interactional model proposed by Egorov and Szabo (2013) by adding and incorporating factors associated with the risk of exercise addiction. The original model was cited over 100 times in less than eight years and, hence, this expansion aims to broaden the research perspectives on how and why (in general) individuals become addicted to exercise.

It is beyond the scope of this review to discuss the original model, which is described in the paper by Egorov and Szabo (2013). Instead, we provide the rationale, relevance, and function for the newly included factors and a new domain. This expanded model yields a more comprehensive picture of the etiology of exercise addiction while prompting further consideration of the ways individuals' exercise habits come to be driven by addictive behavior.

## The expanded interactional model of exercise addiction

**Fig. 2.**
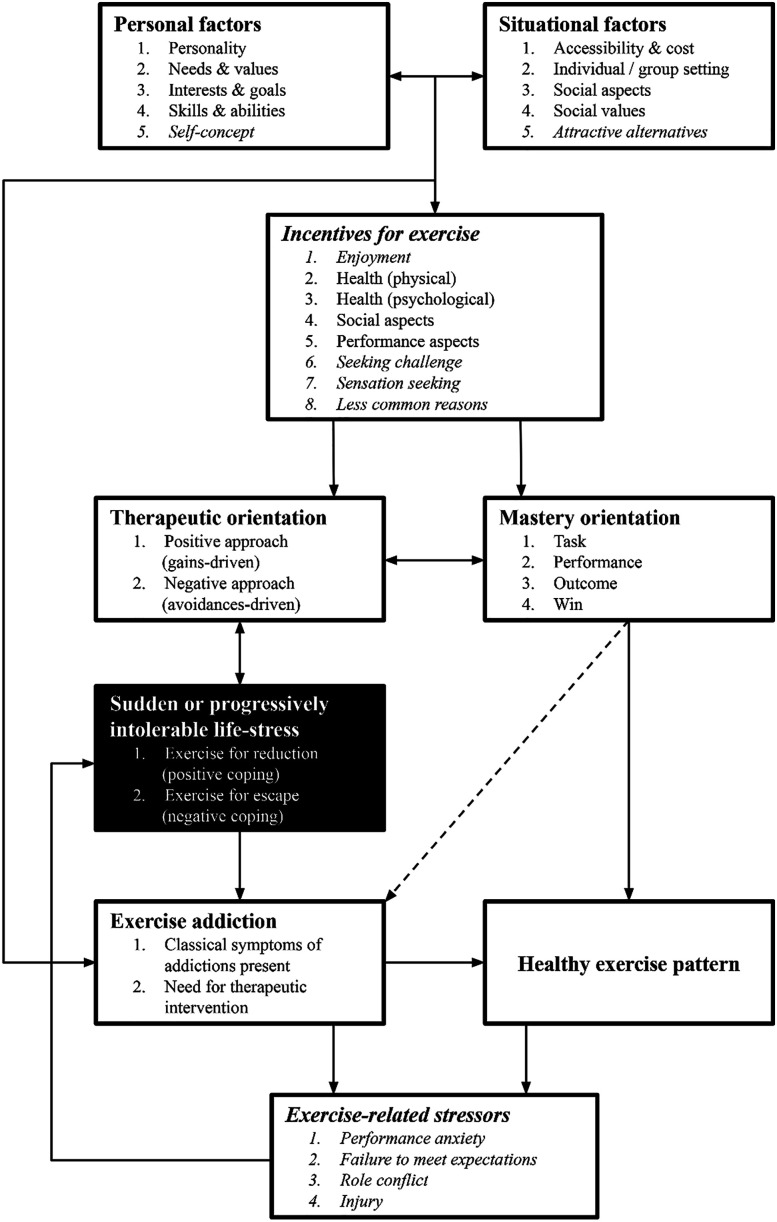
The expanded interactional model of exercise addiction

The expanded model is presented in Fig. 2. It can be easily compared to the original model (Egorov & Szabo, 2013) illustrated in Fig. 1. Modified or added items are in *italics* in the updated model.

Both models include the domains of ‘personal factors’ and ‘situational factors.’ Within these domains, the factors represent personal needs and environmental influences. Although not explicitly stated in the conceptualization of the model, these factors and their interactions follow the basic concepts of the Self-Determination Theory (SDT; Deci & Ryan, 1980; Vallerand, 2000) in which a person's motivation and motivational orientation results from the interaction of personal and environmental factors. The SDT is a valuable framework for understanding motivation in exercise, sports, and training. Indeed, several studies examining exercise addiction have used SDT to explain maladaptive behavioral regulation in both sports and exercise (Edmunds, Ntoumanis, & Duda, 2006; Frederick & Ryan, 1993; González-Cutre, D., & Sicilia, 2013).

### Personal and situational factors

To the domain of ‘personal factors,’ we added *self-concept*, which is a cognitive structure that includes ideas, attitudes, and judgments people use to make sense of the world, focus on their goals, understand their self-worth, and decide which aspects of their self are important (Leary & Tangney, 2012). Self-concept may include elements of identity (i.e., “I am an athlete”) in addition to appraisals of oneself (i.e., “I am out of shape”). This self-construction is relevant in shaping exercise behavior either positively or negatively (de la Vega et al., 2016; Reifsteck et al., 2016; Woolf & Lawrence, 2017).

To the domain of ‘situational factors,’ we have added the item ‘*attractive alternatives.’* This item considers why an individual chooses to engage in sports or exercise instead of other activities; additionally, it can enhance the understanding of why someone prefers a particular form of sports or exercise over others. The situational factors listed in the model resemble those described in the Sport Commitment Model (SCM; Scanlan, Beals, Russell, & Scanlan, 2003). We added *attractive alternatives* as a factor found in SCM, previously not found in the original model, to encompass a broader range of situational factors and make the interactional model compatible with SCM. Although SCM was primarily applied to understand elite athletes' commitment to their sport, it is also beneficial in understanding the values and behaviors of highly committed recreational athletes and exercisers.

### Incentives for exercise

The domain ‘incentives for exercise’ was presented in the original model as ‘exercise motivation’ (Fig. 1). We changed this domain's name primarily for semantic accuracy; these items represent the ways a person seeks to enhance their lived experience, their health, or physical ability, or appearance through participating in exercise and sports. We added four factors to the list, the first of which is *enjoyment*. Enjoyment is often the foremost reason that people exercise (Rodrigues et al., 2020). *Seeking challenge* is another common incentive for exercise and sports participation, particularly among individuals who participate in challenging sports and activities, such as ultradistance running, triathlon, cycling, adventure racing, and other endurance sports (Simpson, Prewitt-White, Feito, Giusti, & Shuda, 2017). People who seek challenges may feel unchallenged in other areas of their lives, disenchanted by mundane routines, or curious about finding their limits. Some individuals may also pursue risky activities to experience certain sensations such as a natural ‘high,’ transcendence, and even suffering (Nogueira et al., 2018). Flow state is yet another sensation or state of being individuals may seek out that can be experienced in different sports and activities (Csikszentmihalyi & Csikzentmihaly, 1990; Zuckerman, 2010). Further, we added the item ‘*less common reasons’*. Whether a person has less common reasons to participate in sports or exercise is highly personal, and examples of these reasons will likely be limited to case studies or examples from popular media such as documentaries, biographies, and personal profiles. Less common reasons for exercise may include strengthening family bonds (Wagoner, 2018), maintaining one's sobriety (Corbett & England, 2018; Wagoner, 2018), managing depression (Busch et al., 2016), supporting charitable causes (Filo, Funk, & O’Brien, 2011), and using sports as a reason for a vacation, traveling, and festival attendance (Green & Jones, 2005). With the addition of the above items, the model now encompasses a much broader range, capturing both the tangible and less tangible incentives a person might seek through exercise.

### Exercise related stressors: A new domain

Finally, we added a new domain of ‘*exercise-related stressors*’ to the model. This domain interacts with ‘sudden or progressively intolerable stress.’ By including this concept, we recognize a significant implication of exercising. Regardless of whether a person's exercise is healthy or driven by addictive behavior, there is potential for them to experience stress due to their involvement with physical activity. Not only is this antithetical to the idea of exercising to relieve stress, but it will also necessitate additional stress-coping responses. For individuals who use exercise to escape from stress, this may result in spiraling further into a pattern of avoidance and escape, creating a dangerous circular causal relationship between physical activity and stress.

The factors we included in this domain are *performance anxiety, failure to meet expectations*, *role conflict*, and *injury*. In this context, performance anxiety is the unpleasant psychological state triggered by a person's perceptions that their capability is being evaluated, causing apprehension and worry (Ford et al., 2017). Performance anxiety may cause stress which can fuel an existing (exercise) addiction. Failure to meet expectations is applicable to the domains of both exercise and sports. When an individual is unable to achieve a desired level of sports performance, achieve a desired body aesthetic, or meet other expectations related to their physical activity they are likely to experience stress associated with failing to realize their desired result. Role conflict occurs when one role makes it difficult to comply with another or when the behaviors involved in one or more roles are incompatible (Tsaur, Liang, & Hsu, 2012). These conflicts arise between the roles a person takes on in sport or exercise and their family, career, education, or other vital roles in their life. The final item in this new domain is injury. Injuries in sports and exercise are common, especially during bouts of focused training, and occur from acute trauma or as the result of overuse. In either case, injuries can be stressful, particularly when they cause interruption to a person's exercise routine. All exercise-related stress sources could fuel the exercise addiction if exercise is the principal means of coping to the affected individual.

## Conclusion

This expansion of the interactional model of exercise addiction by Egorov and Szabo (2013) focuses on aspects of exercise addiction associated with individuals who exercise while taking an even broader perspective on their reasons, expectations, values, and experiences related to participating in sports and exercise. We recognize that exercise and the related domains of sports and training offer a multitude of rewards, some of which are only loosely related, to individuals, and, therefore, people have broadly ranging motives for participating in exercise and sports. This multitude of incentives presents a number of potential pathways a person might come to experience exercise addiction. With this in mind, several additions were made to the original interactional model of exercise addiction to make it more comprehensive, which we view as improving the model's explanatory power.

While more extensive than its initial version, the model still leaves some key questions to be considered. For example, it cannot elucidate how a person's behavior can shift from exercise addiction to a healthy exercise pattern (forms of treatment, changes in personal or situational factors). Potential emotional factors in various domains are unaccounted for in the model. Still, the earlier model has been widely accepted and used in the exercise addiction literature. We hope that the here presented expanded model will be even more helpful.

## Financial support

No financial support was received for this study.

## Author contribution

JD identified the need to revise the interactional model of exercise addiction and wrote the first draft of the paper; AYE verified the theoretical connection between the dimensions of the model and ensured that the expanded model is in agreement with the literature on the topic; AS worked with JD on the expansion of the model and finalized the paper for publication.

## Conflict of interest

The authors have no conflict of interest to declare.
